# Retraction: Constraints on the evolution of toxin-resistant Na,K-ATPases have limited dependence on sequence divergence

**DOI:** 10.1371/journal.pgen.1012195

**Published:** 2026-06-16

**Authors:** The PLOS Genetics Editors

After this article [1] was published, concerns were raised regarding the underlying quantitative assay data available at [2]. Specifically,

- In the IC50 data in the NKA_Enzyme_Assays_Raw_Data file [2] underlying the NKA resistance to CTS results in [1], the Xenodon data in rows 70–77 appears to be the same as the Ostrich and Sandgrouse data in rows 38–45, except for differences in 12 of these reported values.
- In the ATPase Activity data in the NKA_Enzyme_Assays_Raw_Data file [2] underlying the Na,K-ATPase activity results in [1], the Xenodon data in rows 38–40 appear similar to the Ostrich data in rows 22–24.

The corresponding author and the first author SM stated that the above overlapping data regions likely arose due to an error in transferring the underlying data from the original plate reader output files into pre-annotated spreadsheets. They stated that the plates were often read multiple times within a 10 minute window and that several plate reader output windows were open at once. During post-publication discussions, an author also stated that the absorbance readings were manually altered prior to statistical analysis to reduce apparent variances among replicate absorbance readings, raising additional concerns for the reliability and validity of these data.

The *PLOS Genetics* Editors consider that the above data concerns call into question the reliability and integrity of the CTS resistance and enzyme activity results and associated conclusions in [1], and that these concerns cannot be resolved.

In addition to the above concerns, the first author SM stated that the authors had identified an error with the TEG + Q111T protein construct where it contained an unexpected stop codon, and that this could explain the low protein expression of TEG + Q111T reported in Figs S3 and S5 in [1]. They stated the TEG + Q111T experiments had been repeated with the corrected protein construct, and provided updated versions of Figs 5, S4-S6, and Tables S4, S7-S9. In light of the above unresolved concerns for the underlying quantitative data, PLOS did not investigate the concerns for the TEG + Q111T protein construct further.

In light of the above unresolved concerns that call into question the reliability of the reported results and conclusions, the *PLOS Genetics* Editors retract this article.

SM, SHÁ, MdPRO, SD, AJC, and PA agreed with the retraction and stand by the article’s findings. SM apologizes for the issues with the published article. LY, KZ, and JFS either did not respond directly or could not be reached.
